# The Influence of Depression on the Psychometric Properties of the Maslach Burnout Inventory–Human Services Survey: A Cross-Sectional Study With Nursing Assistants

**DOI:** 10.3389/fpsyt.2018.00695

**Published:** 2018-12-18

**Authors:** Telma R. Trigo, Camila C. S. de Freitas, Yuan-Pang Wang, Floracy G. Ribeiro, Mara Cristina S. de Lucia, José O. Siqueira, Dan V. Iosifescu, Jaime Eduardo C. Hallak, Renerio Fraguas

**Affiliations:** ^1^Laboratory of Neuro Imaging (LIM-21), Department and Institute of Psychiatry, Clinics Hospital, University of São Paulo School of Medicine, São Paulo, Brazil; ^2^Section of Psychiatric Epidemiology (LIM-23), Department and Institute of Psychiatry, Clinics Hospital, University of São Paulo School of Medicine, São Paulo, Brazil; ^3^Technical Advisory Office–State Department of Health–São Paulo State Government, São Paulo, Brazil; ^4^Division of Psychology, Central Institute, Clinics Hospital, University of São Paulo School of Medicine, São Paulo, Brazil; ^5^Department of Pathology, University of São Paulo School of Medicine, São Paulo, Brazil; ^6^Department of Experimental Psychology, Institute of Psychology, University of São Paulo, São Paulo, Brazil; ^7^Department of Psychiatry, New York University School of Medicine, New York, NY, United States; ^8^Department of Neurosciences and Behavior, University of São Paulo School of Medicine, São Paulo, Brazil

**Keywords:** burnout, depression, maslach burnout inventory, validity, nursing

## Abstract

**Background:** The Maslach Burnout Inventory-Human Services Survey (MBI-HSS) is the most commonly used instrument to assess burnout. Although various factors have been reported to influence its validity, the influence of major depressive disorder (MDD) has not been previously considered. We developed this study to investigate the influence of MDD on the psychometric properties of the MBI-HSS in nursing assistants.

**Results:** From a sample of 521 nursing assistants, we found in those with MDD (*n* = 138, 24.56%) a degree of data misfit into the model, revealed by non-acceptable values for the root mean square error of approximation (RMSEA; 0.073; *p* = 0.004) and for the comparative fit index (CFI; 0.912), while in the non-MDD group these indices were acceptable and good, respectively, for RMSEA (0.048; *p* = 0.639) and for CFI (0.951). Also, we found higher coefficients of correlation among MBI-HSS factors and less items loading properly in their respective factors in the MDD subset, when compared to the non-MDD subset. For the total sample, while original 3-factor solution was an acceptable model, the bifactor model fitted data better.

**Conclusions:** MDD may impair the construct validity of MBI-HSS subscales, by increasing measurement error and decreasing model fitness. Therefore, researchers and health professionals should be aware of potential changes in the psychometric properties of the MBI-HSS when applied in subjects with depression.

## Introduction

Burnout is a syndrome including emotional exhaustion (EE), depersonalization (DE) and low personal accomplishment (PA), resulting from prolonged stress at work (1–3). The Maslach Burnout Inventory (MBI) is the most widely used instrument to assess burnout; it has been translated into various languages and has been used in innumerous countries (4). Some inconsistencies in its validation and its possible determinants have been described (5–8). Depression has a significant relationship with burnout; however, the potential influence of major depressive disorder (MDD) on MBI validation has not been specifically investigated.

### Background

According to Maslach's description, EE is the syndrome‘s core dimension and refers to feelings of being emotionally overextended and depleted of one's emotional resources (1). DE comprises negative, detached and impersonal attitudes toward other people (i.e., clients and patients). Reduced PA reflects feelings of incompetence at work and negative self-evaluation (1, 9).

Burnout leads to personal suffering including sleep complaints (10), increased use of alcohol/drugs (11), family conflict (12), higher work absenteeism (13), higher staff turnover, early retirement and significant financial impact (14), self-reported unprofessional conduct (15), and suicidal ideation (16, 17).

Nurses play a distinct and critical role in the health system; however they are frequently under increased stress and work overload (18). Burnout is high among nurses and nursing assistants (19–21) in diverse healthcare settings (22–24). Besides the negative impact in the nurse, the overall level of nurse burnout in hospital units has been reported to affect patient satisfaction and other measures of deficient care quality (25). Assessing and minimizing burnout is fundamental to maintain high levels of work performance, decrease nurse shortages (19, 26) and meet recruitment targets for nurses (27).

Several studies using the exploratory and confirmatory factor analysis have validated the MBI in various countries (4–8), including for Brazilian nurses (28, 29). The MBI has a satisfactory reliability, its internal consistency, assessed by the Cronbach's alpha, has been reported to range from 0.70 to 0.90 for its three subscales (30). However, inconsistencies in MBI psychometric properties have been reported (31). For example, relatively low reliability for the DE subscale (32), and a best fit for a four-factor model (6) have been reported. Also, the use of 20 instead of 22 MBI items have been proposed (7, 8). Some factors have been reported to influence the MBI psychometric proprieties including sample characteristics (32) and the presence or absence of burnout (33). Recently, two studies reported the best fit for a bifactor model, composed by a general factor and the three traditional factors (5, 34). In the bifactor model, the items may load simultaneously on a global burnout factor and on the three specific ones (i.e., depersonalization, emotional exhaustion and personal accomplishment). In such approach it is possible to estimate the relevance of the global and the specific dimensions of burnout altogether.

Burnout is highly correlated with depression and an overlap, particularly between EE and depressive symptoms have been highlighted (35). Literature data indicate that burnout partially mediate the relationship between work stress and depression (36), and depression generally follows burnout (37). Emotional exhaustion and depersonalization (38), high levels of psychological demand, low social support at work, and stress due to inadequate work (14) have been shown to be significant predictors of subsequent depression. Importantly, among new graduate nurses, changes in burnout levels have been reported to be accompanied by changes in depressive symptoms and also intention to leave the profession (39). Although they are considered to be distinct entities that complement each other (40), the complexity of their relationship still deserves elucidation.

Depression may potentially influence the validity of a scale, as it has been shown for the Modified Fatigue Impact Scale (41). As above mentioned, some factors have been reported to influence the MBI psychometric properties. Although being highly related with burnout the potential influence of depression on MBI psychometric properties has not been investigated. Thus, we conducted this study with nursing assistants of a university hospital to investigate the influence of depression on the psychometric validity of the MBI-HSS.

## Methods

### Sample

The study was conducted at the Central Institute of the Clinics Hospital, Faculty of Medicine, University of São Paulo (ICHC-FMUSP). According to Burns and Grove, to perform factor analysis for each item of a scale, at least 10 subjects are needed (42). Therefore, to perform the factor analysis of the MBI-HSS, which has 22 items, it would be necessary to study at least 220 individuals. To evaluate the influence of depression on the factor structure of the MBI-HSS, we estimated that depression affects ~20% of subjects with moderate or severe burnout and 7% of subjects with mild burnout (40, 43). Thus, to detect the factorial validity we would need 430 subjects (323 subjects with mild and 97 subjects with moderate or severe burnout) to achieve a test power of 90%, with a two-tailed alpha of 5%. Estimating a rate of missing data of 15%, we estimated that 520 subjects would be necessary. We enrolled 521 nursing assistants belonging to various medical units: 320 individuals working in the general medical unit; 60 in the surgical unit; 66 in the emergency room; and 75 in the intensive care unit. We included 40% of nursing assistants from the day-shift (from 7 a.m. to 7 p.m., *n* = 345) and the night-shift (from 7 p.m. to 7 a.m., *n* = 176) of each unit.

### Data Collection

We performed the interviews during participants' work hours, in offices on their respective units. Two researchers, a psychiatrist (T.R.T.) and a psychologist (C.C.S.) went to each medical unit and invited the nursing assistants consecutively as they met them in the nursing workplace.

### Ethics Statement

The study was approved by the Hospital Committee of Ethics in Research of the Clinics Hospital, Faculty of Medicine, University of São Paulo (CAPPesq–HC-FMUSP); number 1202/07. All participants started the research procedures only after providing signed informed consent.

### Instruments

#### Maslach Burnout Inventory-Human Services Survey (MBI-HSS)

The MBI-HSS is a self-report instrument comprised of 22 items, grouped in 3 subscales. The scores on each subscale are assessed separately: a 9-item subscale for EE, a 5-item subscale for DE and an 8-item subscale for PA. The severity of each item is assessed on a 7-point ordinal scale, based on its frequency from zero (never) to six (always). Cut off points have been established based on the American normative data or dividing the subscales into tertiles or quartiles (44). The Portuguese translation was performed by two psychiatrists; a consensual version was submitted to 5 healthy volunteers from the hospital staff to evaluate its use, feasibility and clarity. The resulting version was back translated by a native English speaker fluent in Portuguese and approved by the author of the original American MBI-HSS version.

#### Primary Care Evaluation of Mental Disorders (PRIME-MD)

A psychiatrist (T.R.T.) used the Portuguese version (45) of the Primary Care Evaluation of Mental Disorders (PRIME-MD) to diagnose MDD. The PRIME-MD is a structured interview divided into five modules comprising the most frequent mental illnesses in primary care (mood disorders, anxiety, somatoform disorders, alcohol problems and eating disorders) (46). The mood module presents 9 yes/no questions investigating the depressive symptoms included in the DSM-III-R criteria to diagnose MDD; these criteria have been preserved unchanged by the DSM-IV and DSM-5 (47).

#### Questions to Obtain Demographic and Occupational Data

A questionnaire was used to collect occupational and demographic data including gender, age, marital status, number of children and professional characteristics (professional experience, weekly working hours and absenteeism over the month prior to the interview–missing days from work). Race was self-reported based on the Brazilian census, including white, black, mixed, Asian, and Indigenous people.

### Statistical Analysis

In the descriptive analysis, we show the absolute frequency and percentages for qualitative variables (gender, race, marital status). Absenteeism was analyzed as a dichotomous variable (by comparing subjects without absenteeism with those absent ≥1 day from work in the month prior to the interview). The number of children were categorized in those with “0,” “1 or 2” and “≥3” children. We report the means and standard deviations for quantitative variables (age, time of working experience in healthcare and weekly working hours). We used the non-parametric Mann-Whitney test to compare numerical variables between MDD and non-MDD subsets.

The severity of MBI-HSS items vary from 0 to 6 in an ordinal range. Therefore, we performed the confirmatory factorial analysis using the weighted least squares means and variance adjusted estimation method (WLSMV) (48), and the software MPLUS 7.4 (49, 50). The CFA was performed for the original 3-factor model and for a bifactor model (51). We tested the bifactor model because of the significant correlation between its subscales and the recent reported good performance of this model for the MBI-HSS (5, 34). To test the validity of construct, we evaluated the fit of the model to the data by the exact fit *p*-value and chi-square/degree of freedom –X^2^/df and the approximate fit with the root mean square error of approximation (RMSEA) and its *p*-value. Considering the X^2^/df, its values indicate good fit if 0 ≤ X^2^/df ≤ 2, acceptable fit if 2 < X^2^/df ≤ 3, and poor if X^2^/df > 3; *p*-values of the X^2^/df indicate good fit if 0.05 < *p* < 1.00, acceptable fit if 0.01 ≤ *p* ≤ 0.05, and values of *p* < 0.01 indicate poor fit. Regarding RMSEA, values 0 ≤ RMSEA ≤ 0.05 indicate good fit, values 0.05 < RMSEA ≤ 0.08 indicate acceptable fit and values of RMSEA > 0.08 indicate poor fit (52); *p*-values of RMSEA indicate good fit if 0.10 < *p* ≤ 1.00, and acceptable fit if 0.05 ≤ *p* ≤ 0.10.

We performed the analysis of practical significance by means of the Tucker-Lewis index (TLI or non-normed fit index–NNFI) and the comparative fit index (CFI). Values of the TLI are considered good if ≥0.97 and acceptable if 0.95 ≤ NNFI < 0.97; values of the CFI are considered good if ≥0.97 and acceptable if 0.97 > NNFI ≥ 0.95 (52).

To test the hypothesis that MDD impairs the performance of MBI-HSS scale, we conducted the analysis for the subset of nursing assistants without MDD (n-MDD) and for the subset with MDD (MDD) separately. We performed the configural invariance analysis for the non-MDD and MDD subsets for the 3-factor model. For the bifactor model, we performed the configural invariance analysis, and also the scalar invariance analysis and the analysis of scalar vs. configural invariances.

Factor loadings were initially analyzed by the significance of their standard factor loadings estimates and the correlations between the 3 sub-dimensions of the 3-factor model and of the bifactor model.

To evaluate the internal consistency of each factor in the 3-factor model, we calculated the Cronbach's Alpha using the SPSS Statistics 24 package. We considered values >0.7 as indicative of acceptable indices. For the bifactor model, we included the Explained Common Variance (ECV), the non-hierarchical omega (total omega) and the hierarchical omega indices. These indices evaluate how well the subscale items measure the latent construct, reflecting the reliability of the instrument. We used a significance level of < 0.05.

## Results

### Sample Characteristics

The sample consisted of 521 nursing assistants, the majority were women (91%), white, married and with children (Table 1). The MDD subset (*n* = 138, 24, 76%) was more likely to include women, with children, with longer working experience in healthcare and with increased absenteeism in the month preceding the interview (Table 1).

**Table 1:** Demographic and occupational characteristics of nursing assistants: total sample, MDD and non-MDD subsets.

**Demographic and occupational characteristics**	**Total sample** **N (%)**		**MDD subset** **n (%)**		**Non-MDD subset** ***n*** **(%)**		***P-value***
**GENDER**							
Female	474	91.0	133	96.0	341	87.0	0.011
Male	47	9.0	5	4.0	42	13.0	
**RACE**							
White	275	52.0	76	55.0	190	49.6	0.446
Mixed	141	27.0	33	24.0	116	30.2	
Black	104	20.0	29	21.0	74	19.3	
Asian	1	1.0	0	0	3	0.8	
**MARITAL STATUS**							
Married	260	50.2	72	52.2	189	49.4	0.567
Single	162	31.1	36	26.0	125	32.6	
Separated, divorced	64	12.7	19	13.9	47	12.3	
Cohabitating	16	3.0	4	2.9	12	3.1	
Widowed	17	3.3	7	5.0	10	2.6	
**NUMBER OF CHILDREN**							
0	164	31.5	34	25.0	127	32.0	0.038
1 or 2	261	50.1	76	55.0	187	48.0	
≥3	96	18.3	28	21.0	77	19.0	
**ABSENTEEISM, DAYS**							
0	410	78.8	95	69.0	312	80.0	< 0.001
≥1	111	21.2	43	31.0	79	20.0	
**Numerical variables**	**Total sample mean (*****SD*****)**		**MDD mean (*****SD*****)**		**Non-MDD mean (*****SD*****)**		***P*****-value**
Age, years	39.5	(13)	39.8	(9.8)	39.2	(9.7)	0.546
Weekly working hours	47.9	(20.6)	45.8	(11)	48.6	(23.2)	0.122
Work experience in healthcare, years	8.5	(6.4)	9.6	(6.7)	8.1	(6.2)	0.018

*MDD, major depressive disorder; Absenteeism, days absent from work in the month prior to the interview; N (%), absolute number (percentage); SD, standard deviation*.

*Significance, P < 0.05*.

Regarding burnout, the MDD subset showed significantly increased scores on EE and DE and decreased scores on PA compared to the non-MDD subset (Table 2).

**Table 2:** Severity of burnout according to the MBI-HSS, total sample, MDD and non-MDD subsets.

	**Total sample** **(*****N*** **=** **521)**		**Non-MDD subset** **(*****n*** **=** **383)**		**MDD subset** **(*****n*** **=** **138)**		***P-value***
**MBI-HSS subscales**	**Mean**	***SD***	**Mean**	***SD***	**Mean**	***SD***	
EE	21.80	12.68	18.97	11.5	29.7	12.6	< 0.001^a^
PA	36.48	6.97	37.04	6.8	34.9	7.3	0.004^a^
DE	5.95	5.52	5.34	5.1	7.64	6.33	< 0.001^a^

*MBI-HSS, Maslach Burnout Inventory-Human Services Survey; EE, emotional exhaustion; PA, personal accomplishment; DE, depersonalization; SD, standard deviation; MDD, major depressive disorder. Significance, P < 0.05*.

a *Mann-Whitney test*.

### Construct Validity

For the total sample, almost all indices were good or acceptable in the bifactor model; in the 3-factor model, the X^2^/df and the RMSEA indices were acceptable, and the CFI and the TLI were below the acceptable cutoff. In the simultaneous analysis (configural), the indices indicated acceptable fit in the bifactor model for MDD and n-MDD subsets, except for CFI and TLI, while in the 3-factor model, acceptable indices were found for the RMSEA only. The scalar analysis for the bifactor model revealed good or acceptable values for all indices, except for the CFI.

In the n-MDD subset, most of the MBI-HSS indices were good or acceptable in the bifactor model (i.e., X^2^/df, RMSEA and its *p*-value, and CFI); in the 3-factor model, acceptable and good indices were found for the RMSEA and its *p*-value, respectively (Table 3). However, in the MDD subset, no index indicated good fit, and acceptable indices were found for X^2^/df and RMSEA in the 3-factor and in the bifactor models (Table 3).

**Table 3:** Factor Analysis of the MBI-HSS.

**Model**	**X^**2**^ / df; *p***	**RMSEA; *p***	**CFI**	**TLI**
**1-FACTOR**				
Total sample (*N =* 513)	1381/209 = 6.61; < 0.001	0.104; < 0.001		
Non-MDD subjects (*N =* 377)	1072/209 = 5.13; < 0.001	0.104; < 0.001	0.784	0.762
MDD subjects (*N =* 136)	506/209 = 2.42; < 0.001	0.102; < 0.001	0.806	0.785
Non-MDD and MDD subjects (*N =* 513)	1391/549 = 2.53; < 0.001	0.077^a^; < 0.001	0.841	0.866
**3-FACTOR**				
Total sample (*N =* 513)	695/204 = 3.41; < 0.001	0.068^a^; < 0.001	0.928	0.918
Non-MDD subjects (*N =* 377)	433/202 = 2.14; < 0.001	0.055^a^; 0.124^b^	0.942	0.934
MDD subjects (*N =* 136)	351/202 = 1.74^a^; < 0.001	0.073^a^; 0.002	0.903	0.889
Non-MDD and MDD subjects (*N =* 513)	961/537 = 1.79^a^; < 0.001	0.055^a^; 0.057^a^	0.920	0.931
Non-MDD and MDD subjects Configural (*N =* 513)	850/408 = 2.08; < 0.001	0.065^a^; < 0.001	0.916	0.905
**BIFACTOR**				
Total sample	484/184 = 2.63; < 0.001	0.056^a^; 0.046	0.956^a^	0.945
Non-MDD subjects (*N =* 377)	346/184 = 1.88^a^; < 0.001	0.048^b^; 0.639^b^	0.960^a^	0.949
MDD subjects (*N =* 136)	318/184 = 1.73^a^; < 0.001	0.073^a^; 0.004	0.912	0.890
Non-MDD and MDD subjects (*N =* 513)	776/518 = 1.50^a^; < 0.001	0.044^b^; 0.939^b^	0.951^a^	0.956^a^
Configural	663/368 = 1.80^a^; < 0.001	0.056^a^; 0.077^a^	0.944	0.930
Scalar	800/514 = 1.56^a^; < 0.001	0.047^b^; 0.814^b^	0.946	0.951^a^
Scalar vs. Configural	223/146 = 1.53^a^; < 0.001			

*MBI-HSS, Maslach Burnout Inventory- Human Services Survey; RMSEA, Root Mean Square Error of Approximation; CFI, Comparative Fit Index; TFI, Tucker-Lewis index (non-normed fit index); df, degree of freedom; MDD, major depressive disorder. Significance at P < 0.05*.

a *Acceptable fit*.

b *Good fit*.

### Factor Loadings

**Figure 1:**
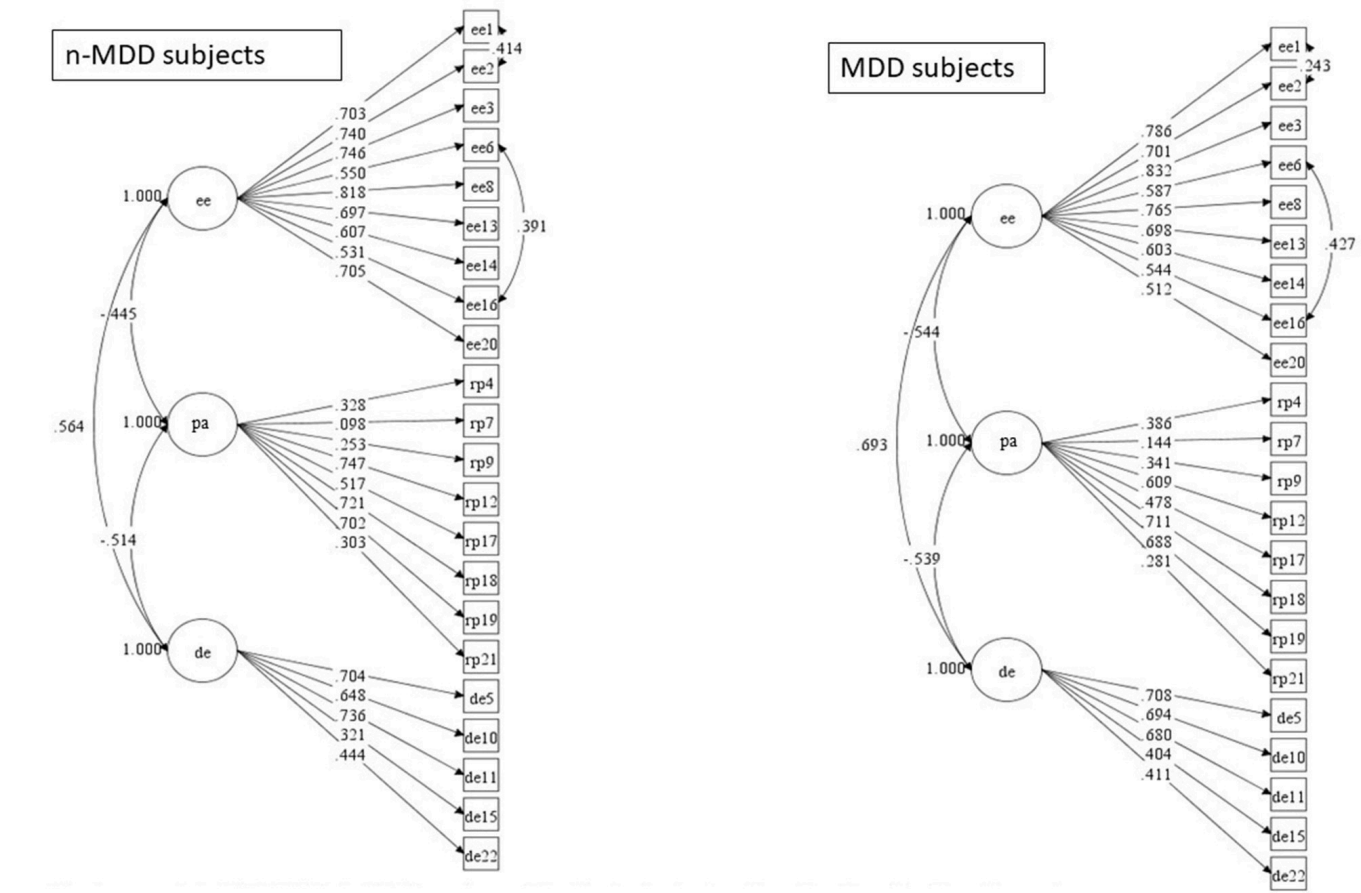
3-factor model of MBI-HSS in MDD and non-MDD subjects including the significant estimate values. MDD, major depressive disorder; EE, emotional exhaustion; PA, personal accomplishment; DE, depersonalization; Non-MDD subset, *N* = 377; MDD subset, *N* = 136.

**Figure 2:**
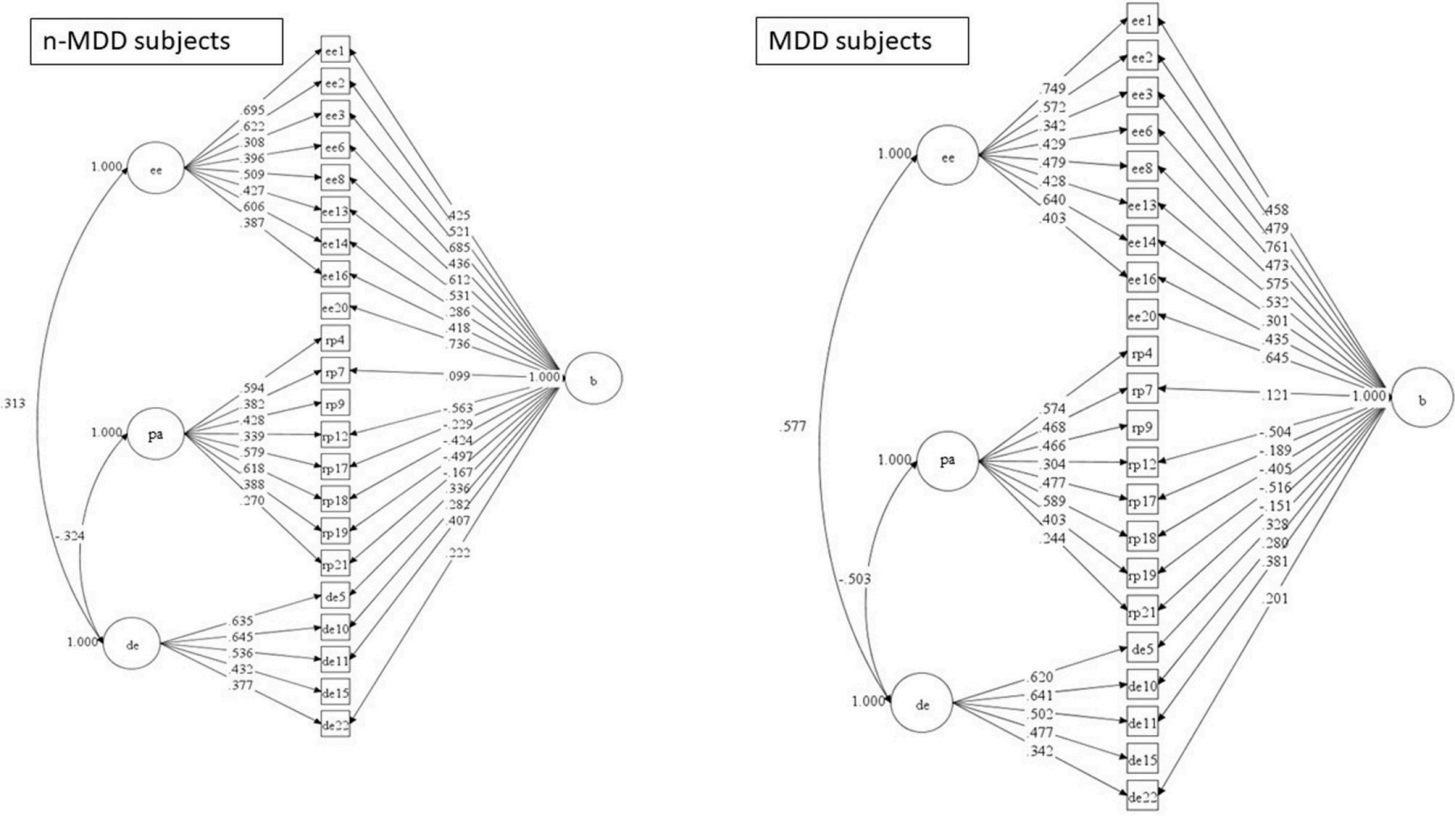
Bifactor model of MBI-HSS in MDD and non-MDD subjects including the significant estimate values. MDD, major depressive disorder; EE, emotional exhaustion; PA, personal accomplishment; DE, depersonalization; Non-MDD subset, *N* = 377; MDD subset, *N* = 136.

In the 3 factors model, all estimates were significant (Figure 1). In the factorial bifactor model, the PA4 (i.e., I can easily understand how my patients feel about things), PA9 (fell positively influencing other people's lives), and the DE15 (don't really care what happens to patients) did not load significantly in the general factor, and the EE 20 (i.e., I feel like I'm at the end of my rope) did not load significantly in the EE factor; both in MDD and in n-MDD subsets (Figure 2).

Considering the cutoff >0.6 as indicative of adequate loading, the n-MDD subset presented more items loading adequate in the bifactor model (i.e., factors EE, PA and in the general factor), and in the 3-factor model (i.e. factor EE) compared to the MDD subset. A similar picture was found for the cutoff >0.5 as indicative of adequate loading; the n-MDD subset showed more items significantly loading in the bifactor model (i.e., EE and PA factors), and in the 3-factor model (i.e., PA factor). No factor showed more items loading >0.6 or >0.5 in the MDD subset compared to the n-MDD subset, both in the bifactor and 3-factor models.

### Correlations

The 3-factor model revealed significant correlations between EE and PA (negative); between EE and DE (positive); and between PA and DE (negative). Significant correlations between EE and DE (positive); and PA and DE (negative) were also found in the bifactor model; however, the bifactor model revealed an absence of correlation between EE and PA both in the MDD and n-MDD subsets. The coefficients of correlation between the subscales were higher in the subset of MDD both for the 3-factor and the bifactor models (Figures 1, 2).

### Internal Consistency

The reliability analysis for the 3-factor model indicated acceptable Cronbach's Alpha index for the EE, both for the MDD and for the non-MDD subsets (Table 4).

**Table 4:** Reliability of MBI-HSS (Cronbach's Alpha): total sample, MDD and non-MDD subsets.

**Factor**	**Total sample**	**Subsets**	
		**Non-MDD**	**MDD**
General	0.724	0.704	0.714
EE	0.868	0.845	0.854
PA	0.587	0.587	0.599
DE	0.551	0.505	0.605

*MBI-HSS, Maslach Burnout Inventory- Human Services Survey; MDD, major depressive disorder; EE, emotional exhaustion; PA, personal accomplishment; DE, depersonalization; Non-MDD subset, N = 377; MDD subset, N = 136*.

Considering the bifactor model, acceptable reliability was found for the total sample and n-MDD subset, but not for the MDD; estimated indices for the non-hierarchical were 0.811; 0.810; and 0.774, respectively, for total sample, non-MDD and MDD subsets. The estimated indices for the explained variance were 0.621; 0.641; and 0.672, respectively, for total sample, non-MDD and MDD subsets. The hierarchical omega revealed that most of the variance was explained by the general factor and only a small proportion of variance was explained exclusively by the EE, PA, and DE individually for the total sample and for both subsets non-MDD and MDD individually (Table 5).

**Table 5:** Reliability of MBI-HSS for the bifactor model (hierarchical omega coefficient): total sample, MDD and non-MDD subsets.

**Factor**	**Total sample**	**Subsets**	
		**Non-MDD**	**MDD**
General	0.575	0.640	0.595
EE	0.094	0.014	0.030
PA	0.105	0.119	0.104
DE	0.038	0.037	0.045

*MDD, major depressive disorder; EE, emotional exhaustion; PA, personal accomplishment; DE, depersonalization; Non-MDD subset, N = 377; MDD subset, N = 136*.

## Discussion

For a sample of 521 nursing assistants at a teaching general hospital, we found that MDD influenced the psychometric properties of the MBI-HSS. Additionally, when considering the total sample, although the original 3-factor model showed an acceptable fit, the bifactor solution provided incremental fitness to the model.

### The Influence of Depression on MBI-HSS Properties

The influence of MDD on the performance of MBI-HSS was revealed by results showing non-acceptable fit indices for the MDD subset and acceptable or good indices for the non-MDD subset (i.e., indices of RMSEA and CFI), and higher correlations among MBI-HSS factors in the MDD subset compared to the non-MDD subset.

Our findings corroborate previous data showing differences on the performance of the MBI, as a consequence of sample psychopathology/characteristics. Schaufeli et al. found good fit indices for the 3-factor model in burned-out employees, but six items of PA did not load accordingly among those non-burned-out (33); indicating a weak support for validity of the PA subscale in those without burnout. Of note, by definition, symptoms in burned-out employees had to be restricted to workplace, while symptoms in those non-burned-out did not have that restriction. Considering that their sample was comprised of employees who were seeking psychological treatment, the non-burned-out group surprisingly had higher psychopathology, including higher levels of depression and anxiety symptoms (33). Consequently, in their sample, the performance of the MBI was worse in the group with increased depression symptoms (i.e., the non-burned-out group), as we found here. Another example for the influence of sample characteristics on the MBI performance comes from workers taking care of individuals with intellectual disabilities. In that sample, Chao et al. found a better fit for a 4-factor solution instead of the traditional 3-factor model (32). In particular, items assessing DE loaded in two DE sub-factors (32). According to the authors, such particularity could be explained by taking into account that services for people with intellectual disabilities are person-centered and focused on building family-like relationships (32). In that situation, the wording in the DE subscale would be unacceptable to those workers, leading to inconsistent responses and impeding items to combine in a single DE factor.

The stronger correlations found in the MDD subset suggest that MDD may influence the MBI structure by decreasing the original conceptually proposed independence of its dimensions. Interestingly, among elementary and secondary teachers, also using the bifactor model for the MBI, depressive symptoms were reported to be related to the general factor of burnout, instead of any specific dimension (34). Our MDD subset also showed less items with factor loadings >0.50 or >0.60 in the PA and DE factors compared to the non-MDD subset both in the bifactor and in the 3-factor models. Thus, our results suggest that the presence of MDD could decrease the strength and the independence of the PA and DE dimensions.

It is possible that depressed mood could affect the subjectivity of the symptom perception and consequently limit the validity of an instrument. Such possibility, was proposed by Larson to explain the influence of depressive mood on the validation of an instrument to assess fatigue (41). According to this thinking, MDD could lead to a distinct interpretation of some MBI items, or possibly a qualitative/quantitative change in experiencing some of them, leading to a diverse scoring and consequently influencing MBI-HSS psychometric properties, as we found in our MDD subset of nursing assistants.

### MBI-HSS Properties for the Total Sample

For the totality of our sample, our results supported the 3-factor model, but indicated a better fit for the bifactor model, confirming the findings reported by Meszaros et al. (5) and corroborating their proposal of including a general burnout dimension in the conceptualization of burnout (5).

We found no correlation between EE and PA in the bifactor model, both in the MDD and n-MDD subsets. This independence of PA has previously been reported (5, 6), reinforcing that PA cannot be interpreted as an opposite of EE and DE (6).

The presence of a general factor in the bifactor model allowed the delineation of the specificity of an item. For example, in the PA factor the items “I can easily understand how my patients feel about things” (i.e., item PA4) and “feel positively influencing other people's lives” (i.e., item PA9) loaded in the PA but not in the general factor both in MDD and in n-MDD subsets (Figure 2). Such findings support the specificity of these items as markers of PA. Of note, in the study of Meszaros et al. the PA4 item also did not load in the general factor (5). In contrast, the item EE20 (i.e., I feel like I'm at the end of my rope) loaded in the general factor but not in the EE factor; both in MDD and in n-MDD subsets (Figure 2), suggesting that it actually assess a general aspect of burnout and not specifically EE.

The reliability of the 3-factor model assessed by the Cronbach‘s Alpha index was higher for the EE than for the DE and PA factors. The higher reliability of EE compared to DE and PA has been reported by previous studies (30, 53) and for samples from various countries (4). Actually, although instruments capturing burnout have included distinct dimensions [e.g., professional repression, dehumanization, emotional distancing (54, 55); enthusiasm toward the job, indolence, guilt (56)], they tend to maintain the exhaustion dimension (54, 55). Those findings support the original view that EE is the burnout syndrome‘s core dimension (1).

In the bifactor model, the non-hierarchical omega index indicated a good reliability, and the hierarchical omega revealed that most of the variance was explained by the general factor. These results converge with the above-mentioned proposal that a general burnout factor should be considered.

Some limitations of our study should be addressed. Our nursing assistants are from a teaching hospital and it is not possible to generalize our findings to settings outside this context. Additionally, our study recruited only in a healthcare setting in Brazil and cannot address whether cultural issues specific to Brazil moderate the impact of MDD on MBI psychometric properties.

## Conclusions

Our data support that the presence of MDD may decrease the construct validity of MBI-HSS, as shown by non-satisfactory values of RMSEA and CFI, and also decrease the independence of its dimensions, as shown by an increase in their correlation, particularly between EE and DE. Such influence may result from distinct interpretation of some MBI-HSS items or from distinct experiences of some burnout symptoms by MDD subjects. Based on our findings, we suggest that researchers consider the influence of MDD when using the MBI-HSS to assess burnout in depressed individuals. We also found a best fit for a bifactor model, including a general factor. To avoid spurious conclusions about MBI-HSS subscales, it is advisable to perform factorial analysis to identify potential influence of the sample, including the influence of depression. Additional studies are warranted to confirm the MDD influence on MBI-HSS validity in different cultures and healthcare settings.

### Relevance to Clinical Practice

In this study, assessing burnout in 521 nursing assistants, we found that a current MDD episode results in a negative impact on the MBI-HSS psychometric properties. Consequently, studies and programs intending to assess and reduce burnout should consider checking MBI-HSS psychometric properties, particularly in those with MDD.

## Author Contributions

TT worked in the idealization of the study, literature search, wrote the protocol, collected the data, worked in the data analysis and wrote the first draft of the manuscript. CF worked in the literature search, collected the data and contributed to the writing of the manuscript. Y-PW worked in the analysis of the data and contributed to the writing of the manuscript. FR worked in the design of the study and worked in the writing of the manuscript. MdL worked in the design of the study and worked in the writing of the manuscript. JS worked in the analysis of the data and performed the statistical procedures. DI worked in the idealization and design of the study and in the writing of the manuscript. JH worked in the rational of the validation process and writing of the manuscript. RF worked in the idealization and design of the study, supervised the data collecting, worked in the data analysis and writing of the manuscript. All the authors contributed and approved the final version of the manuscript.

### Conflict of Interest Statement

The authors declare that the research was conducted in the absence of any commercial or financial relationships that could be construed as a potential conflict of interest.
